# Transcriptome-wide analysis of alternative routes for RNA substrates into the exosome complex

**DOI:** 10.1371/journal.pgen.1006699

**Published:** 2017-03-29

**Authors:** Clémentine Delan-Forino, Claudia Schneider, David Tollervey

**Affiliations:** 1 Wellcome Trust Centre for Cell Biology, University of Edinburgh, Edinburgh, United Kingdom; 2 Institute for Cell and Molecular Biosciences, Newcastle University, Newcastle upon Tyne, United Kingdom; University of Basel, SWITZERLAND

## Abstract

The RNA exosome complex functions in both the accurate processing and rapid degradation of many classes of RNA. Functional and structural analyses indicate that RNA can either be threaded through the central channel of the exosome or more directly access the active sites of the ribonucleases Rrp44 and Rrp6, but it was unclear how many substrates follow each pathway *in vivo*. We used CRAC (UV crosslinking and analysis of cDNA) in growing cells to identify transcriptome-wide interactions of RNAs with the major nuclear exosome-cofactor Mtr4 and with individual exosome subunits (Rrp6, Csl4, Rrp41 and Rrp44) along the threaded RNA path. We compared exosome complexes lacking Rrp44 exonuclease activity, carrying a mutation in the Rrp44 S1 RNA-binding domain predicted to disfavor direct access, or with multiple mutations in Rrp41 reported to impede RNA access to the central channel *in vitro*. Preferential use of channel-threading was seen for mRNAs, 5S rRNA, scR1 (SRP) and aborted tRNAs transcripts. Conversely, pre-tRNAs preferentially accessed Rrp44 directly. Both routes participated in degradation and maturation of RNAPI transcripts, with hand-over during processing. Rrp41 mutations blocked substrate passage through the channel to Rrp44 only for cytoplasmic mRNAs, supporting the predicted widening of the lumen in the Rrp6-associated, nuclear complex. Many exosome substrates exhibited clear preferences for a specific path to Rrp44. Other targets showed redundancy, possibly allowing the efficient handling of highly diverse RNA-protein complexes and RNA structures. Both threading and direct access routes involve the RNA helicase Mtr4. mRNAs that are predominately nuclear or cytoplasmic exosome substrates can be distinguished *in vivo*.

## Author summary

In all organisms, diverse classes of RNA perform a wide range of functions, including programing of protein synthesis, transcriptional and post-transcriptional regulation of gene expression and forming key structural and functional components for pre-mRNA splicing, ribosome synthesis and translation. In consequence, control of the quantity and quality of RNA synthesis and maturation is of key importance. In Eukaryotes, the RNA exosome complex plays a major role in both degradation of aberrant RNA molecules and maturation of functional RNAs. The exosome has a barrel-like structure, with a central channel through which RNAs are threaded to the exonuclease active site of Rrp44. However, in a different exosome conformation, RNAs can also directly access the Rrp44 active site. Here we report the interaction of RNAs with different exosome components and its major cofactor Mtr4 in wild type and mutant strains of yeast. We identified numerous RNAs with clear channel specificities, whereas channel-threaded and direct access routes cooperated for many other substrates. The pathway used by RNA to access Rrp44 reflects the nature and structure of the RNA and whether it is addressed to the exosome for accurate processing or complete degradation, helping us to understand these crucial but highly complex pathways *in vivo*.

## Introduction

In Eukaryotes, the exosome is the major RNA degradation complex responsible for quality control of most transcripts in both the nucleus and cytoplasm, processing of stable RNA precursors and turnover of pre-mRNAs, mRNAs and large numbers of non-coding RNAs (ncRNAs). A puzzling aspect of exosome substrate targeting is the basis of the distinction between precise 3’ processing of stable RNA species and the rapid, complete degradation of “constitutive” degradation substrates or aberrant RNAs and RNA-protein complexes (reviewed in [1]). Processing targets include precursors to the 5.8S rRNA, small nucleolar RNAs (snoRNAs) and small nuclear RNAs (snRNAs). Constitutive nuclear exosome targets include pre-rRNA spacer regions and several hundred different non-protein coding RNAs (ncRNAs). Constitutive cytoplasmic targets are the ~6,000 mRNA species. Aberrant RNAs apparently arise from all classes of transcription unit, including pre-rRNAs, pre-tRNAs, pre-mRNAs and the precursors to many other stable RNA species.

**Fig 1:**
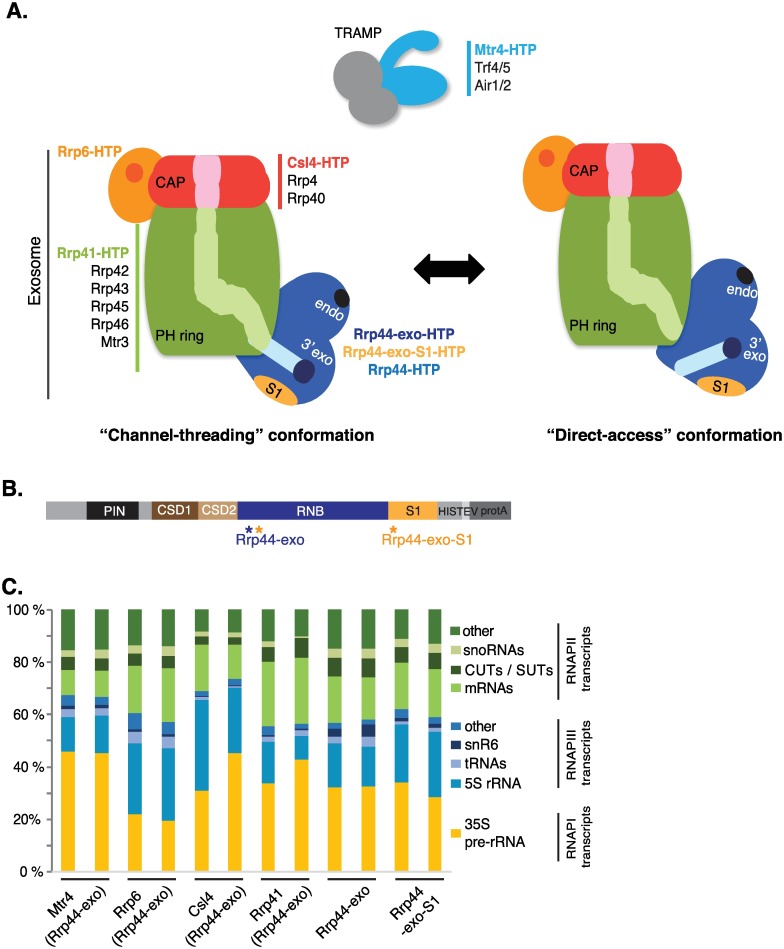
Exosome structure model and interactions. (A) Overview of the structures of the TRAMP nuclear cofactor complex and the exosome. The main components of the exosome are schematically represented: the cap in red, the PH-ring in green, forming a contiguous channel (in lighter color) through which the RNA can be threaded. Active sites are indicated in Rrp6 (orange; exonuclease) and Rrp44 (dark blue; endonuclease (endo) and 3’ ->5’ exonuclease (exo)). The Rrp44 S1 RNA binding domain is represented in yellow and the channel to access the Rrp44 exonuclease site in light blue. Two conformations are illustrated: “channel-threading" of the substrate in which the exosome barrel channel is connected to the Rrp44 channel (left panel). A structural rearrangement can disconnect both channels to allow “direct-access” of substrates to the Rrp44 exonuclase site (right panel). Proteins analyzed by CRAC are in bold color. (B) Domain structure of the Rrp44-HTP fusion. From N-terminus to C-terminus, the following domains are indicated: PIN (PilT N terminus) domain harboring endonuclease activity, CSD (Cold-Shock Domain) RNA binding domain, RNB (RNase II ribonuclease) domain harboring exonuclease activity, S1 RNA binding domain and the HTP-tag (His6, TEV protease cleavage site, protein A). (C) Distribution of reads mapped to different RNA substrate classes recovered in CRAC datasets. Two biological replicates are shown for each protein.

The exosome core has a barrel-like overall structure [2] and in the archaeal complex the nuclease active sites are positioned within a central channel (Fig 1A) [3]. In yeast and human cells the central channel is well conserved compared to Archaea, but point mutations have apparently eliminated the ancestral phosphorolytic activity of the complex [4–6]. Instead, hydrolytic exonuclease activity of the core exosome is provided by an associated protein termed Rrp44/Dis3 in yeast or Dis3 in humans. In the yeast nucleus and human nucleolus, a second exonuclease associates with the exosome, termed Rrp6 in yeast or EXOSC10 in humans.

Rrp44 is composed of an N-terminal PIN domain responsible for endonuclease activity, two continuous RNA-binding cold-shock domains (CSD domains), an RNB domain carrying the exonuclease active site, and an RNA-binding S1 domain (Fig 1B). RNA substrates can reach the exonuclease site by threading through the central channel or via direct access [5, 7–9].

In the major pathway to the exonuclease active site of Rrp44, single-stranded substrates are threaded through the central channel, which protects around 33 nt of RNA [10]. Functional analyses of the PIN domain endonuclease activity of Rrp44 identified only the 7S pre-rRNA and excised 5’ ETS pre-rRNA fragments as targets for cleavage [11–13], whereas UV crosslinking identified apparent interactions between the PIN domain and many exosome substrates [14]. Exosome crystal structures indicate that the PIN domain is not accessed by substrates via the central channel [7]. However, partial occlusion of the central channel by temperature sensitive (ts) mutations in Rrp41 was reported to inhibit both the exonuclease and endonuclease activity of Rrp44 [15]. The exonuclease site in Rrp44 can also be accessed by a more direct route. This involves a structural rearrangement that disrupts the route for RNA through the central channel to the Rrp44 exonuclease site [8, 9]. Mutations in Rrp44 that are predicted to disfavor adoption of this direct access structure were reported to impair the degradation of two characterized exosome substrates, hypomodified tRNA_i_Met and truncated 5S ribosomal RNA (rRNA) [9]. The Rrp44 G916E mutation disturbs the OB-fold (oligonucleotide/oligosaccharide-binding fold) of the S1 RNA binding domain [16, 17] and abolished RNA binding to hypomodified tRNA_i_Met *in vitro* [6].

We anticipated that inactivation of the S1 domain would reduce RNA recruitment by direct access (Fig 1A, right panel), but not via threading through the central channel (Fig 1A, left panel). Conversely, charge-reversal mutations in Rrp41 that impair entry of RNA to the central channel should reduce utilization of the threaded pathway with little impact on direct access substrates. In addition, we anticipated that substrates following a pathway of direct access to Rrp44 might show limited crosslinking to exosome components located in the barrel of the exosome. On RNAs threaded through the channel, we anticipated that the distribution of exosome proteins might be resolved, at least on highly abundant substrates with a well-defined site of stalling.

The extent to which substrates for degradation by Rrp6 pass through the central channel *in vivo*, and in which orientation, remains unclear [18, 19]. However, substrate passage through the channel or, indeed, any interaction with the exosome, is apparently not obligatory for Rrp6 activity. Similar RNA phenotypes are seen following depletion of any of the ten “core” exosome components, including Rrp44, whereas loss of Rrp6 results in distinctly different effects showing that its functions are at least partially independent of the exosome barrel [20, 21]. Many highly structured RNA substrates are preferentially targeted by Rrp6, including the 5.8S+30 pre-rRNA, mature tRNAs, small nuclear (snRNAs) and small nucleolar RNAs (snoRNAs) [14, 22]. The core exosome complex appears to have little activity, but functions *in vivo* with several different activating cofactors in the nucleus and cytoplasm. Key nuclear cofactors include the RNA helicase Mtr4 [23, 24] [25], which can function either alone or in the context of the Trf4/5-Air1/2-Mtr4 polyadenylation (TRAMP) complexes [26–31].

*In vitro* analyses have given major insights into the structure of the exosome and its interactions with RNA and cofactors. However, despite this outstanding work it remains much less clear, *in vivo*, which RNAs and RNA-protein complexes are partitioned between the multiple different routes to the exosome active sites, or whether specific routes are favored for individual substrates that are destined for degradation versus accurate 3’-end processing. The aim of this work was to resolve these questions using a combination of UV-crosslinking and analysis of cDNAs (CRAC) on exosome subunits or cofactors (Mtr4) with mutations in the exosome to identify different classes of directly interacting RNAs.

## Results

UV crosslinking and analysis of cDNA (CRAC) was performed in actively growing cells as described [32, 33] on Rrp44-HTP, carrying a C-terminal, His6-TEV-Protein A tandem affinity purification tag, and on constructs carrying point mutations to inactivate the exonuclease catalytic site (Rrp44-exo; D551N) and disrupt RNA binding by the S1 domain (Rrp44-S1; G916E) as previously reported [6] [14]. We anticipated that lowered RNA binding affinity due to the S1 domain point mutation would reduce recruitment via the direct access route to the Rrp44 exonuclease active site. Growth tests of strains expressing only the mutant forms of Rrp44 (S1A and S1B Fig), showed minor defects in growth of the Rrp44-S1 strain suggesting that the G916E point mutation does not result in substantial mis-folding of Rrp44. More marked impairment was seen in the Rrp44-exo and Rrp44-exo-S1 strains, however, cultures used for CRAC analyses showed robust growth (S1A Fig).

The Rrp44-exo construct recovered substantially more target RNAs than wild type Rrp44, suggesting that bound substrates were being degraded during the extended incubations needed for purification of the RNA-protein complexes. Rrp44-exo had a similar substrate specificity to Rrp44 wild type, but was enriched for substrates normally degraded rapidly and efficiently by the exonuclease activity, such as RNAPII non-coding RNAs and a subset of RNAPIII transcripts (5S rRNA, U6 snRNA and scR1) indicating that they are under-estimated in Rrp44 wild type [14]. We therefore compared RNA recovered with Rrp44-exo-HTP (D551N) and Rrp44-exo-S1-HTP (D551N, G916E), which we anticipated to impair direct access.

In addition, non-tagged Rrp44-exo was combined with tagged forms of other exosome components, Rrp6-HTP, Csl4-HTP, Rrp41-HTP, and the major nuclear cofactor, Mtr4-HTP (see Fig 1A). The aim was to confirm or refute the threading of substrate RNAs through the central channel. Our expectation was that RNAs directly accessing Rrp44 would show limited crosslinking to Csl4 and Rrp41 in the central channel. The relative association of substrates with Rrp44 and Rrp6 gives a further indication of the degradation pathway involved, while association with Mtr4 indicates the participation of the TRAMP complex. Notably, *in vitro* structural data place Mtr4 only at the entrance to the central channel—distant from the direct access pathway [30, 34]. The combination of Rrp44-exo with tagged forms of Rrp6 and Mtr4 did not influence growth rates. However, its combination with tagged Rrp41 or Csl4 significantly increased the time required for cultures to enter exponential growth.

The distributions of target RNAs recovered from different substrate classes are indicated in Fig 1C. As expected, RNA species transcribed by all three RNA polymerases (RNAPI, RNAPII and RNAPIII) were recovered as exosome substrates. For simplicity, these major groups of transcripts will be discussed separately.

### Handover of RNAPI transcripts

**Fig 2:**
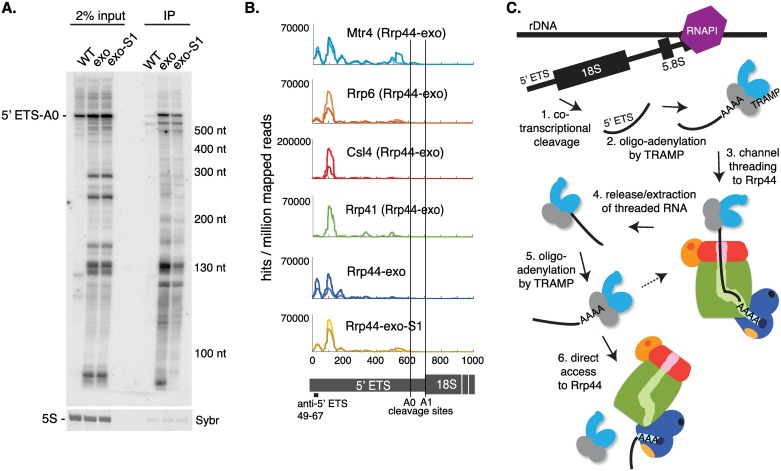
Targeting of the pre-rRNA 5’ external transcribed spacer 5’ ETS (RNAPI transcript) involves both channel threading and channel-independent pathways to access Rrp44. (A) Northern analysis of RNAs coprecipitated with immunoaffinity purified (IP) active Rrp44-HTP (WT), Rrp44-exo-HTP (exo) or Rrp44-exo-S1-HTP (exo-S1), along with 2% input RNA. RNA species are detected with a probe hybridizing near the TSS of the 5’ ETS (+49–67, see panel B for location of the probe). Sybr safe staining for 5S rRNA is shown as loading control. (B) Distribution of reads across the 5’ ETS, recovered with Mtr4, Rrp6, Csl4, Rrp41 in an Rrp44-exo background, and Rrp44-exo and Rrp44-exo-S1, normalized to millions of mapped reads. Scale is linear. A diagram of the 5’ ETS and the 18S rRNA is also shown. (C) Model for 5’ ETS degradation. Following cotranscriptional cleavage of the pre-rRNA, the 5’ ETS is oligo-adenylated by TRAMP and targeted to Rrp44 through the channel. The 5’ ETS is subsequently released from the channel (possibly aided by Mtr4 activity) and subjected to new oligo-adenylation by TRAMP, before being targeted to Rrp44 through direct access.

Pre-ribosomal RNAs are characterized exosome substrates and were enriched in our datasets. In particular, the excised 5’ external transcribed spacer (5’ ETS) that is released by cleavage at site A0 (Figs 2 and S2) and the internal transcribed spacer 2 (ITS2) region that is present on the 7S pre-rRNA (5.8S rRNA with ~140 nt 3’ extension) and 5.8S+30 pre-rRNA (S2 Fig). Notably, both the 5’ ETS and the ITS2 region of the 7S pre-rRNA are also characterized substrate for the endonuclease activity of Rrp44 [11, 13]. It was, however, unclear whether they access Rrp44 directly or via threading.

To address this point, we performed affinity-purification experiments without crosslinking. Rrp44-exo and Rrp44-exo-S1 both coprecipitated the full length 5’ ETS—A0 fragment and multiple degradation intermediates, notably a cluster of bands ranging from ~110–130 nt, indicating threading through the channel to Rrp44 (Fig 2A). However, the shortest fragments detected (~80 nt) using a transcription start site (TSS) proximal probe (+49–67) notably failed to coprecipitate with the exosome lacking S1 RNA binding activity.

Analysis of the read distribution across the 5’ ETS (Fig 2B) showed two major peaks for Rrp44-exo. The peak over +90 to +140 is retained in the Rrp44-exo-S1 double mutant and is also present in the datasets for the Csl4, Rrp41 and Rrp6 exosome components and the TRAMP component Mtr4. Point mutations in the cDNA sequence data indicate the direct sites of protein-RNA contact. Mutation analysis (S2A Fig) indicates that this region contains multiple Rrp44-associated sites, consistent with the multiple bands observed in the northern blot data (Fig 2A). This strongly indicates that the +90 to +140 peak represents a set of major intermediates in Mtr4-mediated degradation by threading to Rrp44, in cooperation with Rrp6. In contrast, the TSS proximal peak (~+20 - +50 nt) in Rrp44-exo was greatly reduced in the exo-S1 double mutant and largely absent from the Rrp41 and Csl4 crosslinking data, and recovered at only a low level withRrp6. It was, however, retained in the Mtr4 crosslinking data. Downstream of the +90 to +140 region, distributed binding is seen in all datasets including Rrp44-exo-S1 and Mtr4. This would be consistent with the northern blot data and indicates largely processive degradation through this region.

The data would support a model in which the region from the 5’ end of the ETS to site A0 (5’ ETS-A0) would be threaded through the channel, contacting Csl4 and Rrp41. We predict that this species is initially submitted to repeated cycles of oligo-adenylation by TRAMP, as previously proposed [26], each facilitating unwinding by Mtr4 and threading (Fig 2C). A number of fragments are detected from this region, which do not appear to be lost in the exo–S1 double mutant. From the location of the +130 nt fragment further degradation may be impeded by strong secondary structure that is predicted in the 5’ domain of the ETS. Short 5’ fragments with 3’ ends matching the major peaks and crosslinks in the ETS around +50 would not be observed in the northern blot analysis. However, the lack of crosslinking to Rrp41, Csl4 or Rrp6 strongly indicates that these RNAs are not threaded though the channel. Despite this, the crosslinking data indicate that this region is a target for Mtr4, strongly suggesting that Mtr4 can also facilitate the direct access pathway to Rrp44.

The 7S pre-rRNA was coprecipitated with similar efficiency by Rrp44-exo and Rrp44-exo-S1 suggesting that is processed by threading through the channel (S2B Fig). However, the shorter 6S pre-rRNA (5.8S rRNA + 5–8 nt 3’ overhang) was less efficiently recovered by Rrp44-exo-S1 than Rrp44-exo. The distribution of reads across the 7S pre-rRNA (S2C Fig) showed a strong peak upstream of the 3’ end of the 5.8S+30 pre-rRNA (indicated by a dashed black line) with all core exosome subunits and, to a lesser extent, with Rrp6. The processing of 7S to 5.8S+30 is normally dependent on the core exosome rather than Rrp6, whereas processing from 5.8S+30 to 6S is strongly Rrp6-dependent [35]. Binding of Rrp44 3’ to 5.8S+30 is not sensitive to inactivation of the S1 RNA binding domain, and this region is also bound by Rrp41, Csl4 and Mtr4, strongly indicating that the 3’ end of the 7S pre-rRNA is threaded through the channel. At 5.8S+30 we speculate that further processing via the channel is blocked by the RNA-protein structure of the mature 5.8S region (see also [36]). The remaining ITS2 region must presumably then be extracted from the channel and re-targeted to Rrp6. The peak of exosome association with the 5.8S+30 region may reflect the time required for this reorganization.

### RNAPIII transcripts show differences in threading through the exosome channel

**Fig 3:**
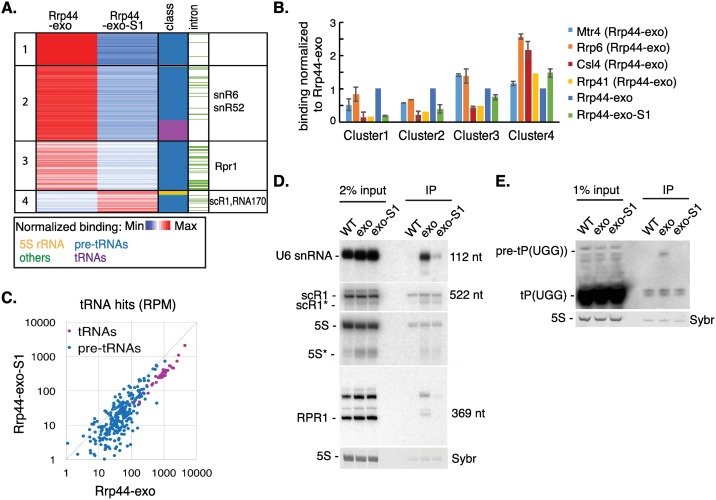
RNAPIII transcripts show differences in threading through the channel. (A) RPMs (reads per million mapped reads) for each RNA species were averaged between two replicates of either Rrp44-exo (column 2) or Rrp44-exo-S1 (column 3) constructs and arranged by k-medians clustering (k = 4, column 1). Distributions of pre-tRNAs (blue), tRNAs (purple), 5S rRNA (yellow) and other non-coding RNAPIII transcripts (green) are indicated in column 4. Intron-containing pre-tRNAs are indicated in green in column 5. Transcripts discussed in the text are indicated in column 6. See also S3 Table. (B) Relative protein association of all RNAPIII transcripts from each cluster was calculated for Mtr4, Rrp6, Csl4, Rrp41, Rrp44-exo and Rrp44-exo-S1 in total RPM. Averages between two independent experiments are shown with standard deviation, except for Rrp41 where fewer reads were recovered and only the largest dataset is shown. (C) 2D scatter-plot comparing RPM across pre-tRNAs and tRNAs recovered with Rrp44-exo and Rrp44-exo-S1. (D-E) Northern analysis of RNAs coprecipitated with Rrp44-HTP (WT), Rrp44-exo-HTP (exo) or Rrp44-exo-S1-HTP (exo-S1), along with 2% (D) or 1% (E) input RNA, probed for RNAPIII transcripts: U6 snRNA, scR1, 5S rRNA, RPR1 (D) or tRNAPro(UGG) (E). Sybr safe staining for 5S rRNA is shown as loading control. Asterisks indicate previously reported truncation products that are known exosome substrates.

The overall distribution of target RNAs (Fig 1C) suggested that binding of RNAPIII transcripts, in particular snR6 and tRNAs, is sensitive to the Rrp44-S1 mutation. To better characterize the interactions of individual RNAPIII transcripts with the channel and direct access pathways, we applied k-means clustering algorithms to the Rrp44-exo and Rrp44-exo-S1 data (Fig 3A). Clustering is based on reads per million total mapped reads (RPM) for each RNAPIII transcript, averaged between two datasets.

Clustering identified four groups of RNAPIII transcripts (Fig 3A). For the large majority of RNAPIII transcripts (Clusters 1–3) interactions with Rrp44 were strongly reduced by the S1 mutation, since substantially fewer reads were recovered with Rrp44-exo-S1 than Rrp44-exo. This indicates that these RNA species predominately access Rrp44 in a channel-independent manner. For Cluster 4 transcripts, relative recovery was higher with Rrp44-exo-S1 compared to Rrp44-exo. In the double mutant many RNAs show reduced binding, so species for which binding is unaltered will show an apparent increase in relative recovery. We conclude that Cluster 4 transcripts are insensitive to loss of the S1 RNA binding activity, indicating that they are predominately threaded. These species included the 5S rRNA, scR1 (RNA component of the signal recognition particle, SRP), RNA170 and a subset of pre-tRNAs.

Most tRNA isoacceptors are transcribed from multigene families in which the mature tRNAs are the same but the flanking, transcribed pre-tRNA regions are unique. Pre-tRNAs were differentiated by aligning sequences to a database composed only of pre-tRNA regions (with 15 nt overhang on each end) and using only uniquely mapped reads. For the mature tRNA regions, the genomic source cannot be differentiated within gene families, and they appear as a single entry in Fig 3A. Reads that match internal tRNA sequences may therefore be generated from mature tRNAs or precursors. However, given the large excess of mature tRNAs, these are likely to predominate and the reads are designated as “tRNA”. In the cluster analysis, mature tRNAs predominately fall in cluster 2, which is characterized by particularly low binding to Rrp44-exo-S1 relative to Rrp44-exo. In contrast, pre-tRNAs and other RNPIII transcripts were distributed between different clusters. A subset of yeast pre-tRNAs contain introns (shown in green in “intron” column of Fig 3A), but these were not clearly segregated from non-intronic pre-tRNAs. Notably, pre-tRNAs for the same isoacceptor did not systematically fall into the same clusters, suggesting they are not processed in the same way.

The RNAPIII cluster data derived from Rrp44 crosslinking was compared to the association of the same transcripts with the exosome channel components, Csl4 and Rrp41, exonuclease Rrp6 and cofactor Mtr4 (Fig 3B). Here, binding to different proteins was averaged between two independent experiments and is presented relative to Rrp44-exo binding (set to 1). For Rrp41 CRAC, which reproducibly recovered relatively low numbers of reads, only the experiment with the highest number of reads was used, to reduce noise in the calculation. Association with Csl4 and Rrp41 was significantly higher for cluster 4, consistent with threading of these substrates through the channel. Conversely, Csl4 and Rrp41 binding was the lowest for cluster 1, in which transcripts are the most affected by the S1 mutation, with intermediate levels for clusters 2 and 3. Association with Rrp6 was strong in all clusters suggesting that a major turnover pathway for tRNAs involves targeting to Rrp6. This is consistent with a previous report that tRNAs show higher binding with Rrp6 than Rrp44 [14].

RNAs from cluster 4 showed more than two-fold higher relative binding by Rrp6 or Csl4 than Rrp44. We speculate that these substrates can be degraded either by Rrp6, which directly contacts Csl4 [18], or by threading through the channel to Rrp44. Association with Mtr4 was seen across all clusters, indicating that it functions as a cofactor in both channel-mediated and direct access to Rrp44. The highest association with Mtr4 was on cluster 3 transcripts, with relative binding similar to Rrp6 and slightly greater than Rrp44-exo. This suggests that these substrates could use either Rrp6 or direct-access to Rrp44 for their degradation, facilitated by Mtr4 in both cases.

The cluster analyses indicated that while pre-tRNAs show some differences in their interactions with Rrp44, mature tRNAs were grouped together in cluster 2 and all were sensitive to the S1 mutation. This was further analyzed by comparing each tRNA and pre-tRNA on a 2D plot (Fig 3C). Mature tRNAs showed markedly decreased binding to Rrp44-exo-S1 relative to Rrp44-exo, suggesting they directly access Rrp44 *in vivo*. Pre-tRNAs showed a much greater spread in their relative interactions with Rrp44-exo-S1 and Rrp44-exo, consistent with the cluster analysis. These data suggest that a subset of pre-tRNAs are threaded, whereas the majority use the direct access pathway.

To independently assess dependence of RNAPIII transcripts belonging to different clusters on the Rrp44 S1 binding domain for exosome association, coprecipitation without crosslinking was performed with Rrp44-HTP, Rrp44-exo-HTP and Rrp44-exo-S1-HTP (Fig 3D and 3E), followed by Northern blotting. The cluster analyses indicated that exosome association of snR6 (U6 snRNA) (cluster 2), RPR1 (RNA component of RNase P) (cluster 3) and pre-tRNA^Pro^_UGG_ (pre-tP(UGG)) (cluster 2, 3, 4) should be dependent on the S1 RBD, whereas binding of 5S rRNA and scR1 (cluster 4) was expected to be less S1-dependent. Northern hybridization confirmed that this is the case, with U6, RPR1 and pre-tP(UGG) showing reduced coprecipitation with the Rrp44-exo-S1 mutant. The 3’ truncated 5S species (5S*) is a well characterized exosome substrate and its binding was not clearly affected by the S1 mutation (see also S3A–S3C Fig).

### Exosome distribution across individual RNAPIII transcripts

We investigated the distribution of protein-binding sites in more detail across individual RNAPIII transcripts. As examples, (S3A–S3C Fig) shows these data for RPR1, snR6 and 5S rRNA. On RPR1, binding of Rrp44, Mtr4 and Rrp6 showed strong accumulation close to the TSS (S3A Fig). Their distribution was similar to the RNAPIII subunit Rpo31 [37] and was not clearly altered in the S1 mutant. This suggests a degree of pausing or stalling leading to release of nascent RNAs that are degraded via the exosome channel. In contrast, a strong peak at the 3’ end of the transcript does not correlate with high RNAPIII occupancy or binding to Rrp41, and the association of this site with Rrp44 is lost in Rrp44-exo-S1. This indicates that post-transcriptional 3’ processing or degradation of RPR1 involves direct access to Rrp44, in cooperation with Mtr4 and Rrp6. The pattern was broadly similar at the 3’-end of the U6 snRNA (S3B Fig) but distinct on the 5S rRNA (S3C Fig; and see above).

**Fig 4:**
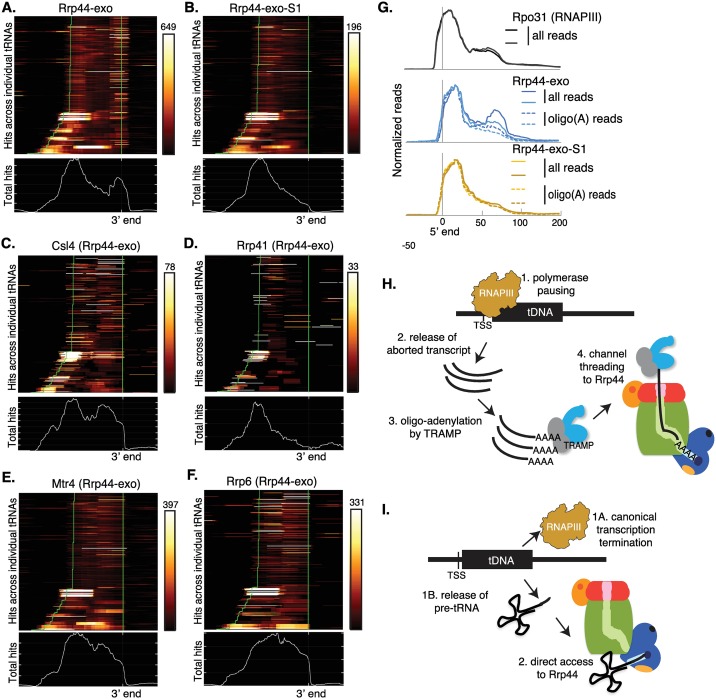
Rrp44 is involved in two distinct tRNA processing pathways. (A-F): Metagene analysis of binding across tRNAs for Rrp44-exo (A), Rrp44-exo-S1 (B), Csl4 (C), Rrp41 (D), Mtr4 (E) and Rrp6 (F), all in an Rrp44-exo background. Upper plots show read distributions across all tRNA genes ordered by length and aligned by the 3’ termini of mature tRNAs. Numbers above color scale box indicates the maximum number of hits recovered in individual genes. Green lines indicate the 5’ and 3’ boundaries of the mature tRNAs. Longer genes at the foot of the columns are intron-containing. Total reads are presented in lower graphs in each panel. (G) Metagene analysis of binding across tRNA genes for Rpo31 (RNAPIII subunit) (black), Rrp44-exo (blue) and Rrp44-exo-S1 (yellow) aligned to mature tRNA 5’ ends. Dashed lines in the Rrp44 samples indicate binding profiles specifically for reads that include 3’ oligo(A) tails absent from the genomic sequence. (H) Model for the degradation of 5’ regions of tRNAs: Aborted transcripts released by RNAPIII pausing are oligo-adenylated by TRAMP and targeted to Rrp44 through the exosome channel. (I) Model of 3’ processing of tRNAs: Following transcription termination, 3’ extended pre-tRNAs directly access Rrp44.

To assess the distribution of protein-binding sites across pre-tRNA and tRNA species, each individual tRNA gene was displayed in 2-dimensional plots (Fig 4A–4F). Each line on the Y axis corresponds to a transcription unit. The X axis shows the absolute position on the gene aligned to the 3’ end of the mature tRNA. Metagene plots below the heat maps show the sum of binding across all individual genes.

Rrp44-exo was strongly bound to both the 5’ and 3’ ends of pre-tRNAs (Fig 4A), indicating that both 5’ and 3’ extended pre-tRNAs are Rrp44 substrates. In comparison, binding of Rrp44-exo-S1 was strongly reduced over the 3’ extended pre-tRNAs and the 3’ region of the mature tRNAs (Fig 4B). The distribution of reads mapped to Rrp41, located within the central channel, closely resembled Rrp44-exo-S1, strongly indicating that (pre-) tRNA 5’ regions are threaded substrates. Distributions of reads recovered with the exosome cap component Csl4, Rrp6 or Mtr4 were similar to each other but distinct from Rrp44, with a sharp drop at the 3’ end of the mature tRNA region (Fig 4D–4F). In summary, this indicates that 3’ ends of pre-tRNAs directly access the Rrp44 active site, while 5’ regions of (pre-) tRNAs are threaded through the central channel. In contrast, mature tRNAs are degraded via a different pathway involving Rrp6, Mtr4 and perhaps Csl4 in the exosome cap. Notably, in published structures, Mtr4 directly contacts Rrp6, which binds to Csl4, and the route taken by substrates to the active site of Rrp6 is likely to involve interactions with the Rrp4/Rrp40/Csl4 ring [19, 30].

Mtr4 is a component of the Trf4/5-Air1/2-Mtr4 polyadenylation complex (TRAMP), which adds oligo(A) tails to RNAs prior to targeting them to the exosome for degradation. Mapping of reads that carry oligo(A) tails that are not encoded in the genomic sequence can therefore identify TRAMP targets. (Pre-) tRNA reads containing non-encoded oligo(A) tails represent ~23% of total reads for both Rrp44-exo and Rrp44-exo-S1, but only ~2% for reads recovered with the RNAPIII subunit Rpo31. Fig 4G presents the alignment of oligo-adenylated reads to tRNAs in comparison with all reads. Notably, the pre-tRNA 3’ regions that are bound by Rrp44-exo but not Rrp44-exo-S1 or Mtr4 also showed low oligoadenylation (Fig 4A, 4B, 4E and 4G), indicating that they are not predominately TRAMP substrates.

Strong binding of Rrp44-exo across pre-tRNA 5’ regions was very similar to Rpo31 (Fig 4G) [37]. RNAPIII pausing or slowing was previously observed over the box A internal promoter region [37] and we speculate that this can result in the release of truncated pre-tRNAs. These are apparently targeted by the TRAMP complex (Fig 4E), oligoadenylated, and reach Rrp44 through the channel, since they are not clearly affected by the S1 mutation (Fig 4G and 4H). Notably, short, truncated RNAPII transcripts are also targeted by the TRAMP and exosome complexes [38–40] (and see below).

For each tRNA, relative protein-binding to the 5’ region versus the 3’ region was calculated. The correlations between the binding profiles obtained for Rpo31 (RNAPIII), Rrp44-exo and Rrp44-exo-S1 were determined by calculating Pearson coefficients (S3D Fig). The total Rrp44-exo-S1 and oligo-A Rrp44-exo-S1 datasets are highly correlated with each other and with Rpo31. Rrp44-exo oligo(A)^+^ reads are substantially better correlated with RNAPIII than were total Rrp44-exo reads. This is consistent with the model that Rrp44 is involved in 2 events: A cotranscriptional (pre-) tRNA degradation pathway, in which TRAMP and the exosome channel play major roles (Fig 4H), and a post-transcriptional processing pathway, in which (pre-) tRNAs directly access Rrp44 (Fig 4I).

### Exosome binding to RNAPII transcripts

**Fig 5:**
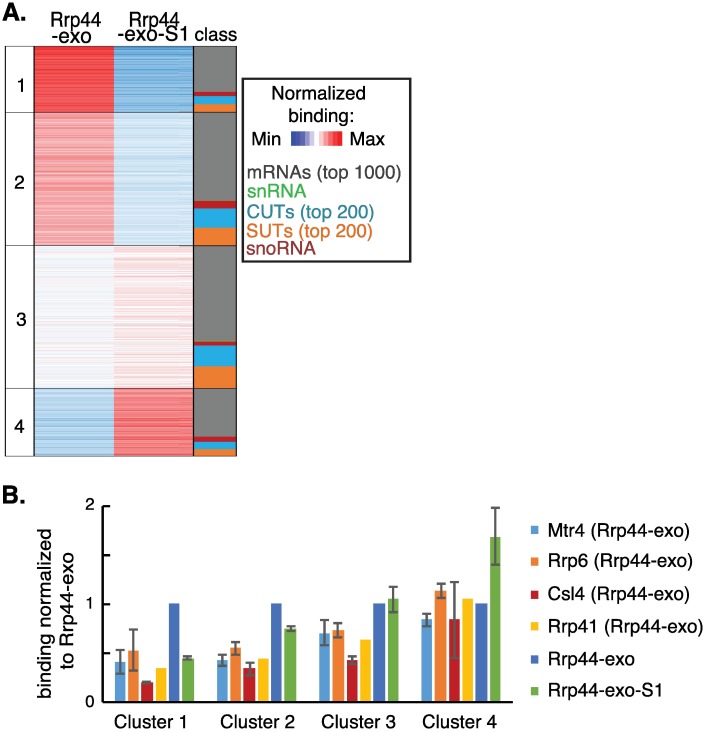
RNAPII transcripts show differences in threading through the channel and direct access to Rrp44. (A) Clustering based on reads per kilobase per million total mapped reads (RPKM) for each transcript for top 1000 mRNAs, top 200 SUTs, top 200 CUTs, 75 snoRNAs and 4 snRNAs. Hits were averaged between two replicates of either Rrp44-exo (column 2) or Rrp44-exo-S1 (column 3) constructs and arranged by k-medians clustering (k = 4, column 1). Location of mRNAs (grey), snRNAs (green), snoRNA (dark red), CUTs (blue) and SUTs (orange) were indicated in column 4. See also S4 Table. (B) Association of all RNAs from each cluster with Mtr4, Rrp6, Csl4, Rrp41, Rrp44-exo and Rrp44-exo-S1 in total RPKM. Averages between two independent experiments are shown with standard deviation, except for Rrp41 where fewer reads were recovered and only the largest dataset is shown.

Clustering analysis was also performed for RNAPII transcripts, allowing us to identify four clusters (Fig 5A). Recovery of Cluster 1 transcripts was strongly reduced by the S1 mutation, indicating predominately channel-independent access to Rrp44. Transcripts in Cluster 2 show a lower degree of dependence on the S1 RNA binding activity. Cluster 3 is enriched for transcripts on which relative binding was unaffected by the S1 mutation indicating that they can use either pathway. For Cluster 4, relative recovery was higher with Rrp44-exo-S1, indicating they are predominately threaded. The distribution of different classes of RNAPII transcripts was broadly similar across the different clusters, indicating that they do not systematically differ in their pathway dependence, in contrast to RNAPIII transcripts.

The relative association of exosome channel components Csl4 and Rrp41 with the different RNAPII clusters correlated with higher relative binding to Rrp44-exo-S1, consistent with increased threading (Fig 5B). Rrp6 and Mtr4 exhibited high binding to cluster 3 and 4 transcripts, suggesting they would play a major role in their degradation, also consistent with the lower sensitivity of these groups to the Rrp44 S1 binding domain mutation. However, we anticipate that mRNAs, in particular, will interact with the exosome and its cofactors in different ways during nuclear pre-mRNA surveillance and cytoplasmic mRNA turnover.

### snoRNA 3’ end processing via direct-access to Rrp44

**Fig 6:**
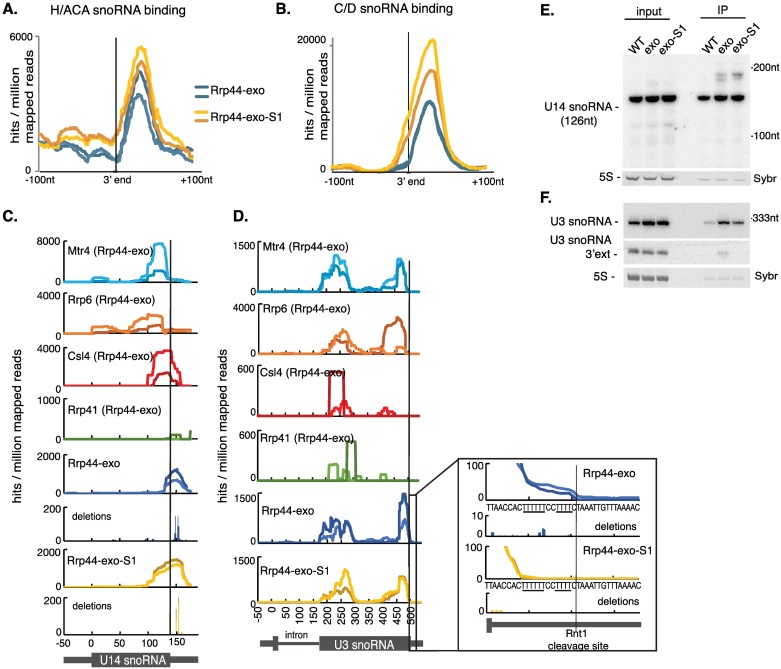
snoRNAs use both channel threading and direct access to Rrp44 for processing. (A, B) Metagene analyses of all box H/ACA snoRNAs (A) or all box C/D snoRNAs (B) aligned by the 3’ end of the mature snoRNA region. Two independent experiments for Rrp44-exo (blue) and Rrp44-exo-S1 (yellow) are shown. (C, D): Distribution of reads across the box C/D snoRNAs U14 (C) and U3 (D), recovered with Mtr4, Rrp6, Csl4, Rrp41 in the Rrp44-exo background, and Rrp44-exo and Rrp44-exo-S1, normalized by millions of mapped reads. Scale is linear. (E-F) Northern analysis of RNAs coprecipitated with wild type Rrp44-HTP (WT), Rrp44-exo-HTP (exo) or Rrp44-exo-S1-HTP (exo-S1), along with 2% (E) or 1% (F) input RNA, probed for the box C/D snoRNAs U14 (E) or U3 (F). Sybr safe staining for 5S rRNA is shown as a loading control.

The box C/D and box H/ACA classes of snoRNA were present in all four clusters (Fig 5A). Alignment of the 3’ ends of all box H/ACA snoRNAs (Fig 6A) shows strong Rrp44 binding downstream of the mature 3’ end, but no significant difference between Rrp44-exo and Rrp44-exo-S1. In contrast, Rrp44 binding downstream of the 3’ end of box C/D snoRNAs (Fig 6B) is elevated in Rrp44-exo-S1, and extends into the mature snoRNA.

Examples of individual box C/D snoRNAs, U14 and U3, are shown in Fig 6C and 6D. Similar read distributions over the 3’ flanking region of the U14 snoRNA were observed for the Rrp44-exo and Rrp44-exo-S1 CRAC datasets (Fig 6C). Single nucleotide deletions (indicating RT errors at the actual site of crosslinking) were mapped to the same nucleotides, showing that both Rrp44 mutants contact U14 at sites 24 nt and 29 nt downstream of the mature 3’ end (indicated by a solid line). Interestingly, even though the contact points are the same for Rrp44-exo and Rrp44-exo-S1, reads are extended further upstream of the 3’ mature end in Rrp44-exo-S1. This suggests that normal processing of pre-U14 involves both direct access and channel threading to Rrp44. Supporting threading, the peak binding sites of Csl4, Mtr4 and Rrp6 lie progressively further upstream of the Rrp44-exo and Rrp44-exo-S1 peaks. Their locations would be consistent with crosslinking to 3’ extended pre-U14 that is threaded through the exosome with a 3’ end located in the Rrp44 active site. RNA coprecipitation (Fig 6E) confirmed that binding of the major 3’ extended form of U14 is not lost when the S1 domain is disrupted, whereas a shorter extended form of U14 was not recovered in association with Rrp44-exo-S1.

In the case of the U3 snoRNA, previous analyses had shown that rapid cotranscriptional cleavage by Rnt1 (RNase III) is followed by binding of the Lsm2-8 complex and Lhp1 (La) to 3’ oligo(U) tracts [41–43]. Rrp44, Mtr4 and Rrp6 all showed binding predominately over the 3’ region of the mature U3 sequence (Fig 6D), whereas little association was observed for the channel proteins Csl4 or Rrp41. Crosslinking of Rrp44-exo also extended into the 3’ flanking region and encompassed the oligo(U) tracts, whereas crosslinking of Rrp44-exo-S1 was limited to the region 5’ to the oligo(U) tracts (Fig 6D; see zoom in). RNA coprecipitation of 3’ extended U3 was lost in the Rrp44-exo-S1 mutant (Fig 6F), indicating direct access to Rrp44. We conclude that this is the major pathway for initial 3’ maturation of U3 snoRNA following Rnt1 cleavage, which is likely to be cotranscriptional. All exosome components, as well as Mtr4, showed additional strong association over the 5’ region of exon 2 of U3. The lack of effect of the S1 mutation plus association with the core exosome components Rrp41 and Csl4 indicates that these U3 regions are threaded through the channel for degradation, possibly of the mature snoRNA.

Overall snoRNA recovery was not strongly affected by the S1 mutation (S4A Fig). The 3’ ends of all snoRNAs were aligned for each of the proteins along the threaded path to Rrp44 (S4B Fig). This revealed the displacement in binding from the top to bottom of TRAMP-exosome complex: Mtr4 occupied the most upstream position, followed by Csl4 and Rrp41, with Rrp44 most downstream. These results indicate that snoRNA turnover mainly proceeds via the threaded pathway. In principle, a similar distribution of factors might be expected on other threaded substrates, however, this is only clearly resolved on strongly expressed substrates with a well-defined site of exosome stalling, such as that predicted to be induced by the snoRNA-associated proteins.

### mRNAs are predominately threaded through the exosome

Binding of the top 1000 expressed mRNAs to Rrp44-exo and Rrp44-exo-S1 was compared (S5A Fig). Their distribution close to the diagonal showed that they are predominately threaded through the channel. On protein-coding genes, Rrp44-exo showed a pronounced peak of TSS proximal binding and this profile was not clearly affected by loss of S1 RNA binding (S5B Fig). Among the few mRNAs with strongly reduced recovery in Rrp44-exo-S1 was *INO1*, which encodes Inositol-3-phosphate synthase [44, 45]. Rrp44-exo binding was strikingly high immediately upstream of the start codon of the gene, whereas these reads were lost completely in Rrp44-exo-S1 (S5C Fig). Other exosome subunits showed few if any hits across *INO1*. The *INO1* gene has a 5’ UTR region of 437 nt, which is exceptionally long for yeast. This highly unusual structure suggests that the region is involved in regulation, and we speculate that direct access to Rrp44 might contribute to the regulation of *INO1* mRNA levels.

**Fig 7:**
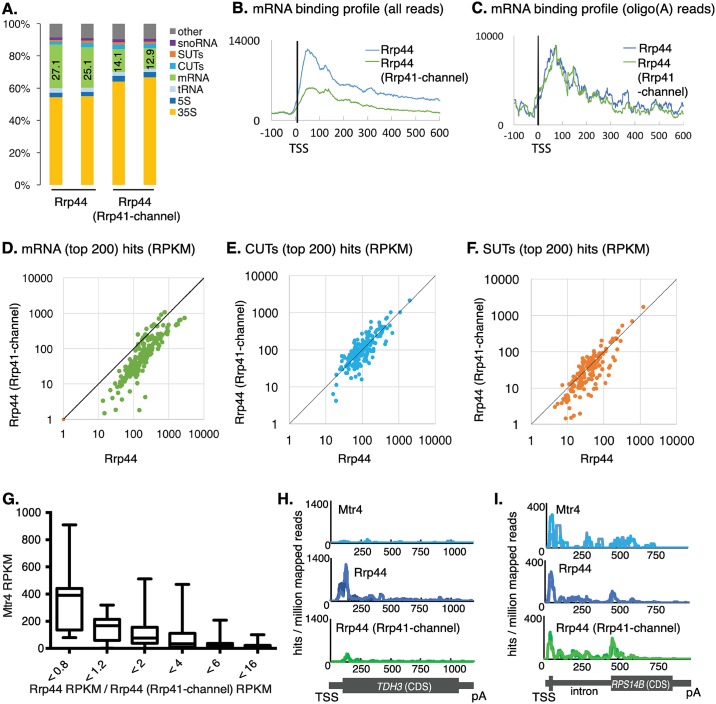
Cytoplasmic processing of mRNAs is affected by the Rrp41-channel mutation. (A) Distributions of RNA classes in mapped reads recovered with Rrp44 in strains expressing wildtype Rrp41 (left columns) or the Rrp41-channel mutant that is predicted to partially occlude the central channel of the exosome (-channel, right columns). Two biological repeats are shown for each strain. (B-C) Metagene analysis of binding across mRNA genes for Rrp44 in strains expressing wild type Rrp41 (blue) or the Rrp41-channel mutant (green) aligned to the transcription start site (TSS), for all reads (B) or only reads that include non-encoded 3’ oligo(A) tails (C), normalized per millions mapped reads. Data from two biological repeats were averaged for each analysis. (D-F) RPKMs for each RNA species were averaged between two replicates of either Rrp44 with wild type Rrp41 or the Rrp41-channel mutant construct and displayed on a 2D scatter plot for top 200 mRNAs (D), top 200 CUTs (E) or top 200 SUTs (F). Species above the diagonal line are predicted to be strongly subject to nuclear degradation. See also S5 and S6 Tables. (G) Mtr4 binding (RPKM) across mRNAs in function of ratio of Rrp44 binding between strains expressing wild type Rrp41 and the Rrp41-channel mutant. Mtr4 preferentially binds mRNAs not affected by channel mutation, consistent with nuclear degradation. (H-I) Distribution of reads recovered with Mtr4 or Rrp44 (with wild type Rrp41 or the Rrp41-channel mutant) across the *TDH3* gene (H), targeted less in Rrp41-channel strains, and *RPS14B*, which is not sensitive to channel mutation, normalized to millions of mapped reads. Scale is linear.

The Rrp44-exo exosome complex protects some 33 nt of RNA *in vitro* [10]. However, this protection is lost in complexes that include a mutant form of Rrp41 with four reverse-charge point mutations at the RNA entry site (K62E, S63D) and exit site (R95E, R96E) of the channel [10]. In an attempt to further define substrates reaching Rrp44 through the channel, we performed CRAC on wild type Rrp44-HTP in a strain where Rrp41 carries these four point mutations (Rrp41-channel). Unexpectedly, comparison of wild type Rrp44-HTP with the Rrp44-HTP, Rrp41-channel strain showed clear crosslinking differences only for mRNAs (Fig 7A), with overall mRNA binding substantially reduced by channel mutation.

In the crystal structure of an Rrp6-associated nuclear exosome complex, the channel was seen to be widened relative to the complex lacking Rrp6 [18]. We therefore postulate that the Rrp41-channel mutation may inhibit RNA passage through the cytoplasmic exosome, but not through the widened channel in the nuclear complex. This model is in agreement with our conclusion that only cytoplasmic exosome substrates, which are predominately mRNAs, are affected by the Rrp41-channel mutation.

Analysis of the distribution of Rrp44 binding over the top 1000 expressed mRNAs showed that the TSS proximal peak of association was reduced, but not abolished by the Rrp41-channel mutation (Figs 7B, S5D and S5E). This 5’ peak was previously attributed to the presence of truncated transcripts generated through premature transcription termination [38–40]. These transcripts are also strongly bound by the nuclear TRAMP polyadenylation complex, and are expected to be degraded in the nucleus. TRAMP substrates are characterized by non-templated oligo(A) tails and we therefore filtered Rrp44 hits for the presence of 3’ oligo(A) tracts. Strikingly, crosslinking of the oligo(A)^+^ transcripts showed no reduction in the Rrp41-channel mutant (Fig 7C). This supports the model that reduced Rrp44 association in the Rrp41-channel mutant is a specific feature of cytoplasmic, but not nuclear exosome substrates.

For each mRNA species as well as the CUT (Cryptic Unstable Transcripts) and SUT (Stable Unannotated Transcripts) classes of ncRNAs, we determined the ratio of Rrp44 binding in Rrp41 and Rrp41-channel strains as a measure of sensitivity to channel narrowing (Fig 7D–7F). Comparison of binding of the nuclear-specific exosome cofactor Mtr4 with mRNA sensitivity to channel narrowing revealed a striking correlation (Fig 7G–7I). Those mRNAs that were least affected by Rrp41-channel (ratio <0.8) were highly bound by Mtr4, whereas mRNAs with Rrp44 association that were strongly reduced in the channel mutant showed progressively decreased Mtr4 association. As examples, hit distributions along two mRNAs are presented (Fig 7H and 7I). *TDH3*, encoding the glyceraldehyde-3-phosphate dehydrogenase, showed almost no binding for Mtr4 or Rrp44 (Rrp41-channel), whereas Rrp44 alone was robustly bound, strongly indicating cytoplasmic turnover (Fig 7H). In contrast, *RPS14B* was targeted by Mtr4 and by Rrp44 in both Rrp41 wild-type and Rrp41-channel strains. Coupled with the recovery of hits across the intron, this indicates that both spliced and unspliced forms of *RPS14B* pre-mRNA can be degraded in the nucleus (Fig 7I).

Based on this insight, mRNAs were analyzed to identify species for which Rrp44 binding was insensitive to the Rrp41-channel mutation, indicating predominant nuclear degradation (Figs 7D, S5D and S5E). This identified a subset of nuclear-degraded mRNAs (above line in Fig 7D). Notably, these include mRNAs encoding four factors implicated in nuclear pre-mRNA surveillance, Nrd1, Nab3, Hrp1 and, most strikingly Dbp2 (S5 Table) [39, 46–49]. Moreover, Dbp2 and Nrd1 are auto-regulated by nuclear RNA processing [50, 51], and this may also be the case for Nab3 and Hrp1. *NRD1* expression is auto-regulated through transcription termination and degradation by the exosome, induced by Nrd1 binding. The peak of Rrp44 binding on *NRD1* corresponded well with the locations of Nrd1-binding sites [52] and consensus Nrd1-binding motifs (UGAUG) (S5F Fig). The mRNA encoding the transcription factor Tye7 is regulated by nuclear RNA surveillance [53] and was also strongly affected (S5 Table).

The CUT class of ncRNAs are well characterized as nuclear exosome substrates and showed little sensitivity to the Rrp41-channel mutation (Fig 7E). In contrast, members of the more stable SUT class of ncRNAs showed an intermediate behavior between mRNAs and CUTs, with different species showing either increased or reduced Rrp44 association in the Rrp41-channel mutant (Fig 7F and S6 Table).

We conclude that the Rrp41-channel mutation offers a tool to distinguish nuclear and cytoplasmic sites of degradation for major exosome substrates.

## Discussion

Here we have attempted to understand how the hugely complex and diverse population of exosome substrates interact with the complex in living cells. This has allowed us to infer clear preferences in pathway to the exonuclease active site in Rrp44.

A difficulty in interpretation of the data is that a single RNA species may form multiple different interactions with the exosome and its cofactors, either during the course of processing/degradation or under different circumstances. As an example, pre-tRNAs and tRNAs showed evidence for at least three types of interaction: 1) Direct-access to Rrp44, involving the TRAMP complex, during degradation or processing of the pre-tRNA 3’ trailer. 2) Channel threading to Rrp44 for truncated TSS proximal pre-tRNA fragments, possibly resulting from premature transcription termination. Analysis of the distribution of the Rpo31 subunit of RNAPIII on tRNA genes showed strong peaks of occupancy, most notably over the box A internal promoter region, which we interpreted as reflecting slowed transcription elongation [37]. The close similarity between the binding of Rrp44 and RNAPIII over tRNA 5’ regions strongly suggests that slowed or stalled RNAPIII has a tendency to release the nascent transcripts, which are bound by TRAMP and targeted to the central channel. Notably, on RNAPII genes short, truncated transcripts are also produced by aborted transcription and targeted by for degradation by the TRAMP and exosome complexes. 3) Degradation of mature tRNAs by direct access to Rrp44, with a major role played by Rrp6. We speculate that the latter activity reflects the tRNA retrograde pathway that can return tRNAs to the nucleus, potentially exposing them to nuclear surveillance.

In our data the 5S rRNA, as well as the truncated 5S* rRNA, appear to be threaded, whereas a recent report [9] proposed its direct access to Rrp44 based on accumulation of the truncated 5S* in a strain expressing Rrp44 mutants that disfavor the direct access conformation. This may further underline the functional redundancy in the degradation system.

On RNAPI substrates, exchange takes place between threading and direct access pathways to Rrp44 on the 5’ ETS, whereas exchange from threading to Rrp6-mediated degradation occurs during 5.8S rRNA 3’ processing. Notably, these transitions require ~30 nt of the substrate to be removed via the “top” of the exosome channel and then to be delivered to the direct access channel of Rrp44 at the “bottom” of the complex, or to Rrp6 at the top. On pre-rRNAs and other substrates we saw peaks of exosome crosslinking at putative sites of handover, which may reflect the time required for this process. The mechanism(s) involved in RNA extraction and retargeting remain unclear, but the helicase activity of Mtr4 is a plausible candidate, and it appears that re-adenylation by TRAMP occurs during handover (see Fig 2C). Degradation of both the 5’ ETS and ITS2 regions was previously reported to involve the endonuclease activity of Rrp44 [11–13]. Since the double exo- endo- mutant is inviable, it is difficult to assess whether the endonuclease activity provides the “backup” to the exonuclease on specific substrates. However, the loss of both activities is lethal, presumably demonstrating the existence of shared, essential substrates.

A striking feature of the data was the effect and apparent specificity of Rrp41-channel mutants, carrying four charge reversal amino acid substitutions that were reported to impede binding of RNA to the central channel *in vitro* [10]. Unexpectedly, strains expressing only Rrp41-channel did not show a clear growth phenotype, and predominately showed changes only in interactions with mRNA, suggesting the mutation is compensated *in vivo*. It was reported that the channel undergoes widening in Rrp6-associated exosome complexes *in vitro* [18]. Our data strongly indicate that the Rrp41-channel mutation has little, if any effect on substrate channeling in the nuclear exosome, but strongly impairs cytoplasmic mRNA association with Rrp44. These results clearly support the model that channel widening occurs *in vivo* and is a distinctive feature of the nuclear exosome.

Published structural data revealed that Mtr4 binds an interface between Rrp6 and Rrp47, while Rrp6 is anchored to the exosome core via a C-terminal extension that binds Csl4 and stabilises the latter in the threaded RNA binding pathway [30]. This location on the “upper” face of the exosome is fully consistent with insertion of substrates into the channel. Unexpectedly, however, Mtr4 appeared to also function together with the direct access route to Rrp44. This strongly predicts the existence of alternative complexes, in which Mtr4 and/or the TRAMP complex will dock with the “lower” face of the exosome to promote loading through the direct access route to Rrp44. It was notable that the direct-access pathway was more important in the nucleus relative to cytoplasmic mRNAs. We speculate that the cytoplasmic Ski2-Ski3-Ski8 complex may be unable to direct RNA to Rrp44 via direct access. This may be a consequence of interactions with the translating ribosome [54] and/or docking exclusively via Ski7, which is bound close to the upper face of the exosome in a position similar to that occupied by Rrp6 in the nuclear complex [55, 56].

Based on the analyses of nuclear oligo(A)^+^ reads, we infer that cytoplasmic Rrp44 shows a clear 5’ peak in mRNA binding. This is in marked contrast to the peak of Ski2 binding previously observed over the 3’ UTR regions of mRNAs [39]. We speculate that the Ski complex is recruited to 3’ UTRs prior to the commencement of mRNA degradation, as seen for other turnover factors [57], and subsequently recruits the exosome following de-adenylation. As the exosome approaches the 5’ cap structure, the presence of the cap-binding proteins may impede the process of RNA threading into the exosome, or the substrate may be less efficiently threaded once it is too short for the Ski complex to remain bound 5’ to the exosome.

### Conclusions

The central channel of the nuclear exosome is widened *in vivo* relative to the cytoplasmic complex, presumably reflecting allosteric changes induced by Rrp6 binding [18]. In consequence, Rrp41 charge-reversal mutations inside the channel [10] inhibited the degradation only of cytoplasmic (non-Rrp6 associated) substrates. This allowed us to distinguish mRNAs and ncRNAs that are preferentially degraded in the nucleus and cytoplasm. In the nucleus, our data implicate Mtr4 in targeting substrates to both the threaded and direct-access pathway, strongly indicating interactions with the exosome that have yet to be observed *in vitro*. Many RNA species showed clear preferences for either the threaded or direct-access pathway. However, these generally appeared not to be absolute requirements. We speculate that this redundancy reflects the outcome of selective pressure. The exosome degrades and/or processes thousands of different substrates, including large numbers of ncRNAs that tend to change rapidly during evolution. The system may therefore have been selected for versatility and redundancy to allow the efficient handling of highly diverse RNA-protein complexes and RNA structures.

## Methods

### Experimental methods

#### Strains

Strains used in this work are listed in S1 Table.

#### CRAC

CRAC was performed as described [32] on yeast strains expressing the protein of interest tagged with a C-terminal His6-Tev cleavage site-Protein A tag, grown in SD-medium to log phase and UV-crosslinked (254 nm, 100 sec) to covalently bind RNA to protein. RNA-protein complexes have been purified, RNAs are partially digested to leave only the “footprint” of the protein or protein complex. Mircat linkers and barcoded linkers (containing three random nucleotides) were ligated on the 3’ and 5’ ends, respectively. Proteins were digested with proteinase K; RNAs were reverse transcribed and PCR-amplified. cDNAs libraries were size-fractionated on agarose gels then subjected to next-generation sequencing using Illumina Hiseq (Edinburgh Genomics) or Illumina Miniseq (our laboratory).

#### RNA coprecipitation

To allow purification of exosome complexes and associated RNA substrates, plasmid-expressed wild type or mutant Rrp44 proteins carried C-terminal fusions with a tag containing the streptavidin-binding peptide, TEV cleavage site and two copies of the z-domain of protein A (szz-tag) or the C-terminal HTP tag used for CRAC analysis (see above and Fig 1B). For each preparation, 500 ml of yeast culture was grown in SD medium at 30°C to OD_600_ ~0.5 and complexes were purified on IgG sepharose in buffer TMN150 (50 mM Tris/HCl pH 7.8, 150 mM NaCl, 0.1% NP-40, 1.5 mM MgCl_2_). To extract the coprecipitated RNA, immobilized protein-RNA complexes were treated with GTC:Phenol:Chloroform for 10 minutes at 65°C. RNA was then separated by acrylamide gel electrophoresis, analyzed by Northern blotting using 5’ end labelled oligonucleotide or random primed labelled probes listed in Supplementary S2 Table and visualized using a PhosphorImager.

### Sequence data analysis

#### Pre-processing and alignment

Sequencing data were quality filtered and adapters were trimmed using Flexbar [58] with parameters –at 1 –ao 4 and only reads containing the 3’ adapter were retained. Then, the sequences were collapsed: reads having identical ends and identical random nucleotides in the 5’ barcode were counted as one, allowing removal of PCR duplicates. For all alignments except tRNAs, sequences considered as low complexity (reads having more than 75% of their content corresponding to a single nucleotide stretch and that would be potentially misaligned) were filtered out. Reads were then aligned to the *Saccharomyces cerevisiae* genome (SGD v64) using Novoalign (Novocraft) with genome annotation from Ensembl (EF4.74) [59], supplemented with non-coding sequences as described in [39], with parameters –r Random, -r Unique or -r All.

#### Pre-tRNAs versus mature tRNAs analysis

To differentiate pre-tRNAs from mature tRNAs (Fig 3A and 3C), processed reads were aligned to a database made only from tRNA genes with 15 nt extensions on both sides of each gene. Uniquely mapped reads were counted as pre-tRNAs, while random mapped reads were counted as a population enriched for mature tRNAs.

#### Clustering

Downstream analyses were performed using pyCRAC software [48].

pyReadCounters (pyCRAC) was used to count overlaps with genes and reads per millions per kilobase (RPKM). For each transcript, we averaged numbers of reads (reads per millions for RNA Polymerase III transcripts, RPKM for RNA polymerase II transcripts) between replicates to reduce the influence of experimental variation upon clustering analysis. For mRNAs, counts between transcription start site and polyadenylation site were counted. For others classes (except tRNAs), a flank of 50 nucleotides around the ends was added to each gene. To account for differences in transcripts abundance and to reflect the relative binding of a transcript to either Rrp44-exo or Rrp44-exo-S1, we normalized the data for each gene. Transcripts were then clustered using Cluster3.0 (k-medians, k = 4, Euclidian distance; Figs 3A and 5A). The data were then displayed as heat maps. For proteins not included in the clustering analysis (Mtr4, Rrp6, Csl4, Rrp41), sums of reads from transcripts of each cluster were calculated and relative binding of each protein across each cluster was displayed as a histogram (Figs 3B and 5B).

#### Plots, binding profiles

Plots showing binding along single genes were generated using pyPileup (pyCRAC). The distribution of reads along transcripts of different classes was performed using homemade scripts using pyPileup on each individual transcript to count hits at each position. Each row represents a transcript and each column represents the absolute position from the 5’ end or 3’ end. The plot sums up binding at each position allowing the display of a binding profile aligned either at the 5’ end or 3’ end.

#### Non-encoded oligo-(A)-tailed analysis

Pre-processed reads containing non-encoded oligo-(A) tails were identified using a pipeline developed by Grzegorz Kudla [39, 60] and aligned as described above.

#### Correlation of binding

pyBinCollector (pyCRAC) has been used on tRNAs. Each tRNA was divided in 2 bins, and hits in each bin were calculated for each dataset either for all reads or reads containing non-encoded oligo-(A) tails for Rrp44 samples. Binding across tRNA halves (5’ or 3’) was calculated as a fraction of total binding across individual tRNAs (set to 1). Averages between two biological repeats were used to calculate Pearson correlation between samples.

## Availability of data and materials

All sequence data are available from GEO under accession number GSE90647.

## Supporting information

S1 Fig Growth tests of strains used in this study. (A-B) *rrp44Δ* strains expressing the indicated HTP-tagged Rrp44 constructs were grown at 30°C in SD -Leu medium for plasmid maintenance to exponential phase, diluted to OD_600_ 0.1 and grown either in liquid media (A) or serially diluted (1:10) and plated (B) in the same medium. (C) *rrp41Δ* strains expressing the indicated Rrp41 constructs were grown and plated on YPD media supplemented with nourseothricin antibiotic (Nat) for plasmid maintenance.
(TIF)

S2 Fig Processing of 7S pre-rRNA to 5.8S rRNA involves both exosome channel threading and direct access to Rrp44. (A) Distribution of reads and deletions across the 200 first nucleotides of 5’ ETS of pre-rRNA, recovered with Mtr4 (Rrp44-exo), Rrp44-exo and Rrp44-exo-S1. Normalized to hits per million mapped reads. Region from +80 to +90 nt is shown in grey. Scale is linear. (B) Northern analysis of pre-rRNAs coprecipitated with active Rrp44-HTP (WT), Rrp44-exo-HTP (exo) or Rrp44-exo-S1-HTP (exo-S1) and 2% of the input RNA, with probe #020 indicated at the bottom of panel C. Sybr safe staining for 5.8S and 5S rRNA is shown as a loading control. Alternative processing pathways operating in the ITS2 region give rise to long and short forms of the mature 5.8S rRNA, designated 5.8S_S_ and 5.8S_L_. (C) Distribution of reads across 7S pre-rRNA, recovered with Mtr4, Rrp6, Csl4 and Rrp41 in the Rrp44-exo background, and Rrp44-exo and Rrp44-exo-S1. Normalized to hits per million mapped reads. Scale is linear. The cartoon shows the 7S pre-rRNA, consisting of the 5.8S rRNA and the ITS2 spacer to cleavage site C1. The 3’ end positions of the 5.8S+30 and 6S pre-rRNAs and mature 5.8S (site E) are indicated together with the probe location use in panel B.
(TIF)

S3 Fig RNAPIII transcripts show differences in their access to Rrp44. (A-C) Distribution of reads across RPR1 (RNase P) (A), U6 snRNA (B) and 5S rRNA (C) recovered with Rpo31 (RNAPIII subunit), Mtr4, Rrp6, Csl4 and Rrp41 in the Rrp44-exo background, and Rrp44-exo and Rrp44-exo-S1. Normalized to hits per million mapped reads. Scale is linear. (D) Pairwise Pearson coefficient of binding across tRNAs. Each tRNA was divided in two bins (corresponding to 5’ and 3’ halves of tRNA) and the number of hits in each bin was calculated for Rpo31, Rrp44-exo and Rrp44-exo-S1. For Rrp44, separate analyses were performed for all reads or only on reads containing non-encoded oligo-(A) tails. Binding across each bin was calculated as a fraction of total binding across individual tRNAs (set to 1). Averages between two biological replicate for each protein were used to calculate Pearson correlations.
(TIF)

S4 Fig snoRNAs are mostly threaded in the exosome channel to access Rrp44. (A) RPKMs for each snoRNA species were averaged between two replicates of either Rrp44-exo or Rrp44-exo-S1 datasets and displayed on a 2D scatter plot. Box C/D and box H/ACA snoRNAs are represented in red and blue respectively. (B) Metagene analyses of all snoRNAs aligned by the 3’ end of the mature snoRNA region. Mtr4 (light blue), Csl4 (red), Rrp41 (green) in Rrp44-exo background, Rrp44-exo (blue) are shown. An average of two experiments was used for each sample, except for Rrp41 in which fewer reads were recovered and only the largest dataset is shown.
(TIF)

S5 Fig mRNAs are preferentially threaded through the channel to access Rrp44. (A) RPKMs for each mRNA species were averaged between two replicates of either Rrp44-exo or Rrp44-exo-S1 datasets and displayed on a 2D scatter plot. (B) Metagene analyses of binding to top 1000 mRNAs aligned by TSS for Rrp44-exo (blue) and Rrp44-exo-S1 (yellow). Two independent experiments are shown for each analysis, normalized per million mapped reads. (C) Distribution of reads recovered with Rrp44-exo and Rrp44-exo-S1 across the INO1 gene, normalized by millions of mapped reads. Scale is linear. (D-E) Metagene analyses of binding of top 200 mRNA aligned by the TSS (D) or poly(A) site (E) for Rrp44 (blue) and Rrp44 (Rrp41-channel) (green). Data from two biological repeats were averaged for each strain background and represented as a fraction of total binding of Rrp44 across mRNAs for each strain. (F) Distribution of reads recovered with Rrp44, Rrp44 (Rrp41-channel) and Nrd1 [52] on *NRD1*, normalized by millions of mapped reads. Scale is linear. Locations of the consensus Nrd1-binding motifs (UGAUG) are also indicated.
(TIF)

S1 Table Strains used in this study. (XLSX)

S2 Table Oligonucleotides used in this study. (XLSX)

S3 Table Composition of RNAPIII transcripts cluster. RPMs (reads per millions mapped reads) for each RNA species were averaged between two replicates of either the Rrp44-exo or Rrp44-exo-S1 construct and arranged by k-medians clustering. Distributions of transcripts among clusters displayed in Fig 3 are listed in this table.
(XLSX)

S4 Table Composition of RNAPII transcripts cluster. RPMs (reads per millions mapped reads) for each RNA species were averaged between two replicates of either the Rrp44-exo or Rrp44-exo-S1 construct and arranged by k-medians clustering. Distributions of transcripts among clusters displayed in Fig 5 are listed in this table.
(XLSX)

S5 Table Rrp44 and Rrp44 (Rrp41-channel) RPKM for mRNAs. RPKMs for each of the 200 most expressed mRNAs are displayed for the two replicates of Rrp44 in strains also expressing either wild type Rrp41 or the Rrp41-channel mutant, and the ratio between average binding of Rrp44 and Rrp44 (Rrp41-channel) was calculated. mRNAs with higher relative binding to Rrp44 in combination with Rrp41-channel are predicted to be strongly subject to nuclear degradation and are listed at the top. See also 2D scatter plot for the top 200 mRNAs (Fig 7D).
(XLSX)

S6 Table Rrp44 and Rrp44 (Rrp41-channel) RPKM for SUTs. RPKMs for each of the 200 most expressed SUTs are displayed for the two replicates of Rrp44 in strains also expressing either wild type Rrp41 or the Rrp41-channel mutant, and the ratio between average binding of Rrp44 and Rrp44 (Rrp41-channel) was calculated. SUTs with higher relative binding to Rrp44 in combination with Rrp41-channel are predicted to be strongly subject to nuclear degradation and are listed at the top. See also 2D scatter plot for top 200 SUTs (Fig 7F).
(XLSX)
