# Supplementary figures and images for: Transcriptome-wide analysis of alternative routes for RNA substrates into the exosome complex
Source: PLoS Genet. 2017 Mar 29;13(3):e1006699. doi: 10.1371/journal.pgen.1006699 (PMC5389853; doi:10.1371/journal.pgen.1006699)

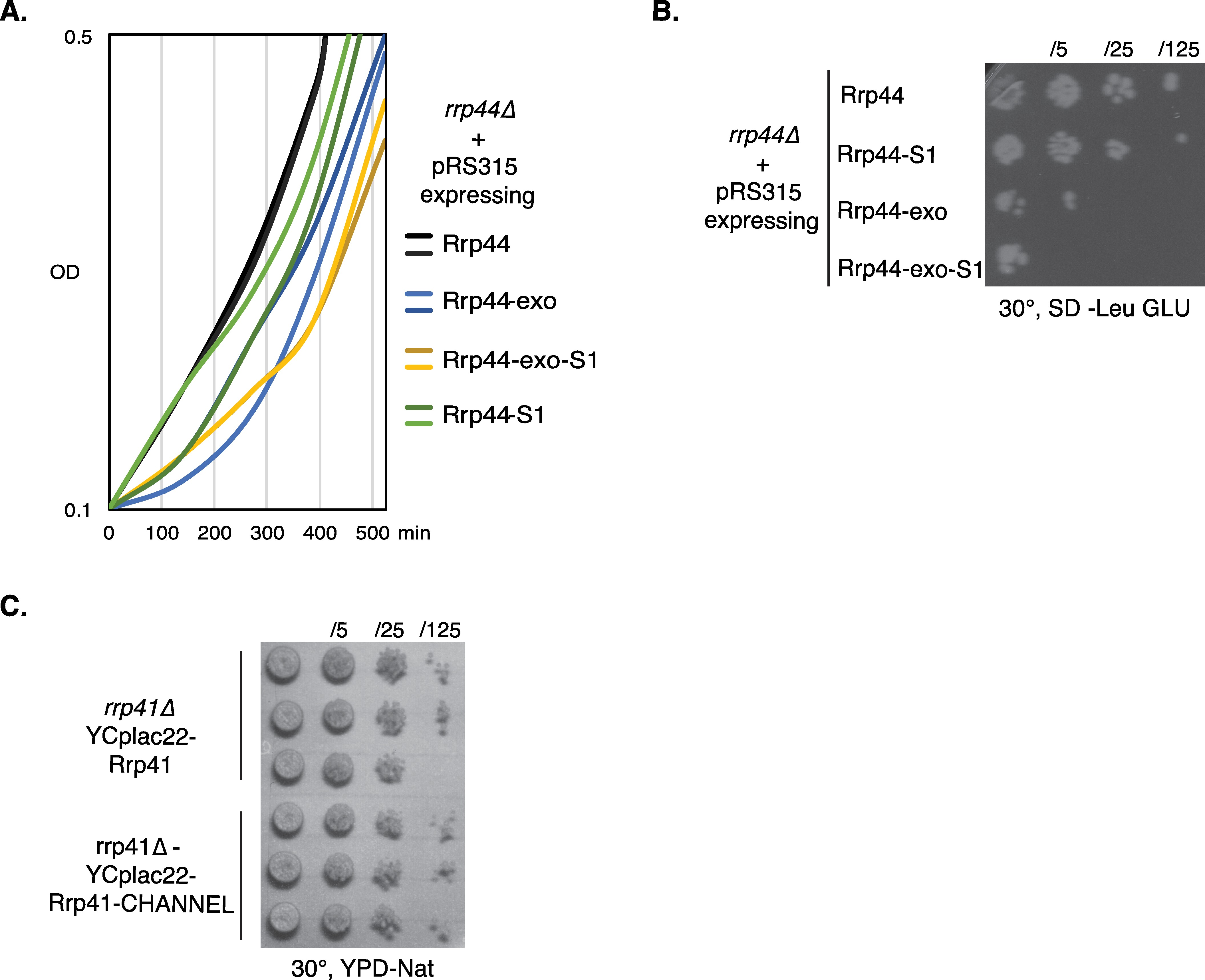

Supplement: S1 Fig — (A-B) rrp44Δ strains expressing the indicated HTP-tagged Rrp44 constructs were grown at 30°C in SD -Leu medium for plasmid maintenance to exponential phase, diluted to OD600 0.1 and grown either in liquid media (A) or serially diluted (1:10) and plated (B) in the same medium. (C) rrp41Δ strains expressing the indicated Rrp41 constructs were grown and plated on YPD media supplemented with nourseothricin antibiotic (Nat) for plasmid maintenance. (TIF) [file pgen.1006699.s001.tif]

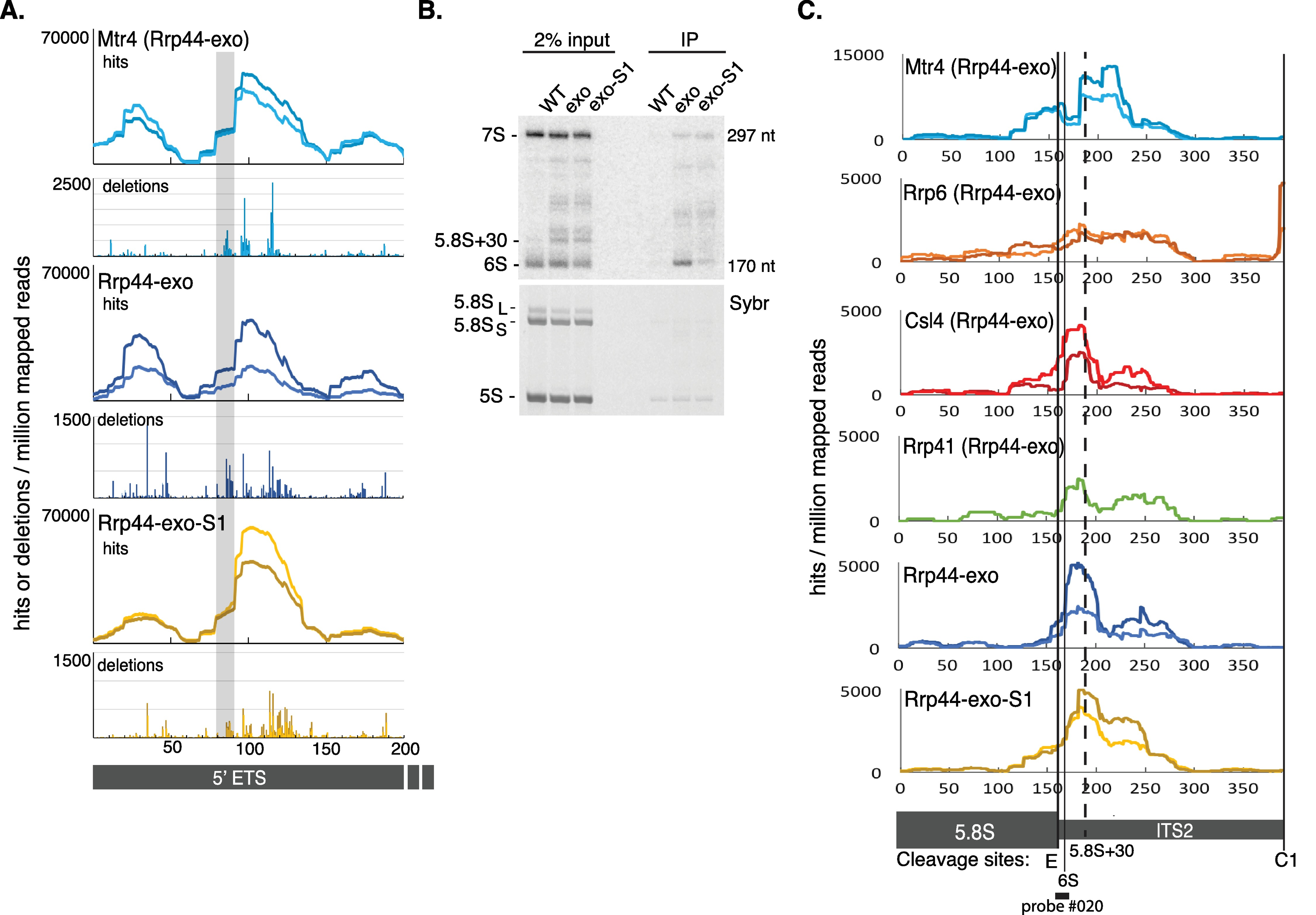

Supplement: S2 Fig — (A) Distribution of reads and deletions across the 200 first nucleotides of 5’ ETS of pre-rRNA, recovered with Mtr4 (Rrp44-exo), Rrp44-exo and Rrp44-exo-S1. Normalized to hits per million mapped reads. Region from +80 to +90 nt is shown in grey. Scale is linear. (B) Northern analysis of pre-rRNAs coprecipitated with active Rrp44-HTP (WT), Rrp44-exo-HTP (exo) or Rrp44-exo-S1-HTP (exo-S1) and 2% of the input RNA, with probe #020 indicated at the bottom of panel C. Sybr safe staining for 5.8S and 5S rRNA is shown as a loading control. Alternative processing pathways operating in the ITS2 region give rise to long and short forms of the mature 5.8S rRNA, designated 5.8SS and 5.8SL. (C) Distribution of reads across 7S pre-rRNA, recovered with Mtr4, Rrp6, Csl4 and Rrp41 in the Rrp44-exo background, and Rrp44-exo and Rrp44-exo-S1. Normalized to hits per million mapped reads. Scale is linear. The cartoon shows the 7S pre-rRNA, consisting of the 5.8S rRNA and the ITS2 spacer to cleavage site C1. The 3’ end positions of the 5.8S+30 and 6S pre-rRNAs and mature 5.8S (site E) are indicated together with the probe location use in panel B. (TIF) [file pgen.1006699.s002.tif]

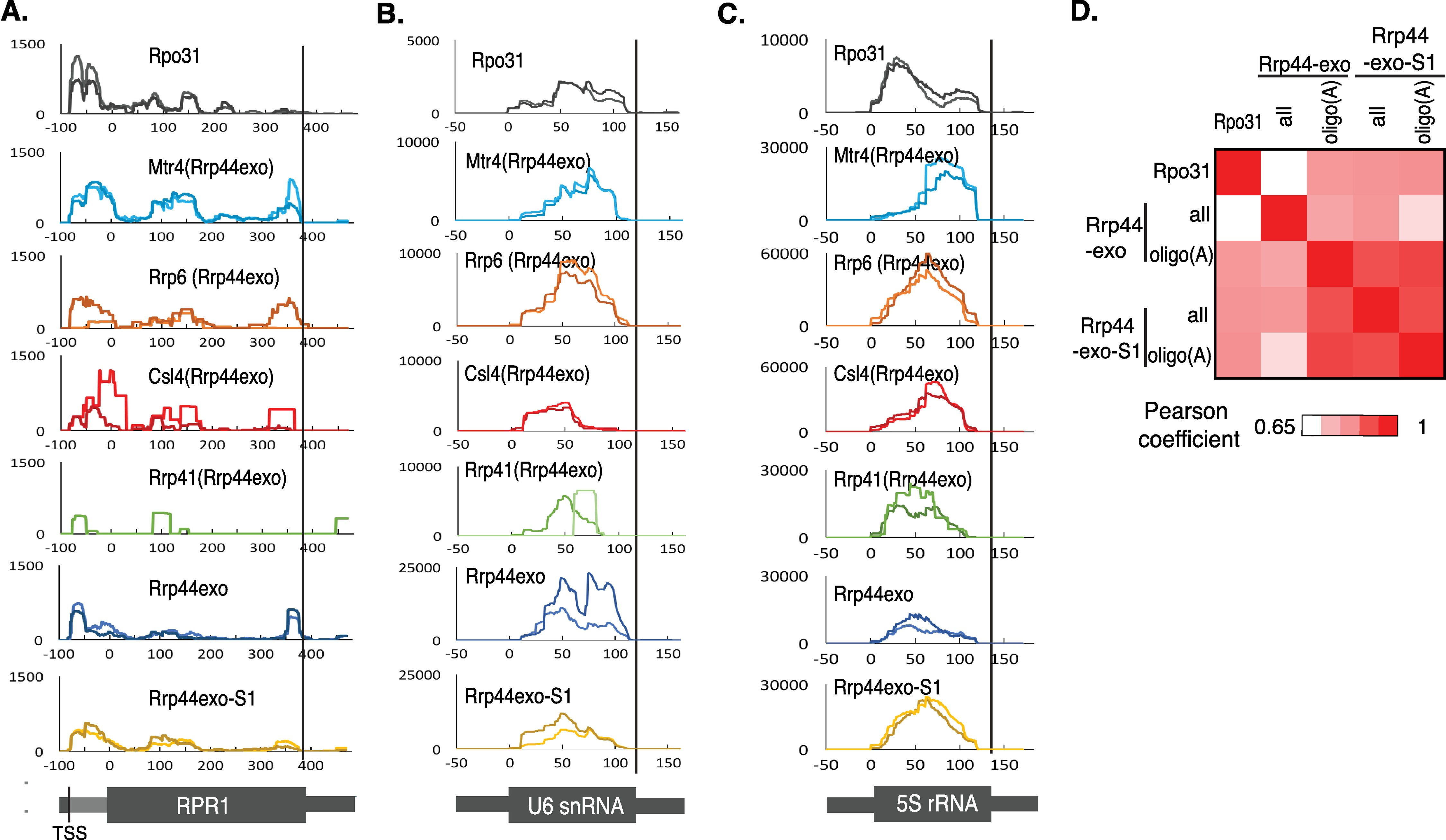

Supplement: S3 Fig — (A-C) Distribution of reads across RPR1 (RNase P) (A), U6 snRNA (B) and 5S rRNA (C) recovered with Rpo31 (RNAPIII subunit), Mtr4, Rrp6, Csl4 and Rrp41 in the Rrp44-exo background, and Rrp44-exo and Rrp44-exo-S1. Normalized to hits per million mapped reads. Scale is linear. (D) Pairwise Pearson coefficient of binding across tRNAs. Each tRNA was divided in two bins (corresponding to 5’ and 3’ halves of tRNA) and the number of hits in each bin was calculated for Rpo31, Rrp44-exo and Rrp44-exo-S1. For Rrp44, separate analyses were performed for all reads or only on reads containing non-encoded oligo-(A) tails. Binding across each bin was calculated as a fraction of total binding across individual tRNAs (set to 1). Averages between two biological replicate for each protein were used to calculate Pearson correlations. (TIF) [file pgen.1006699.s003.tif]

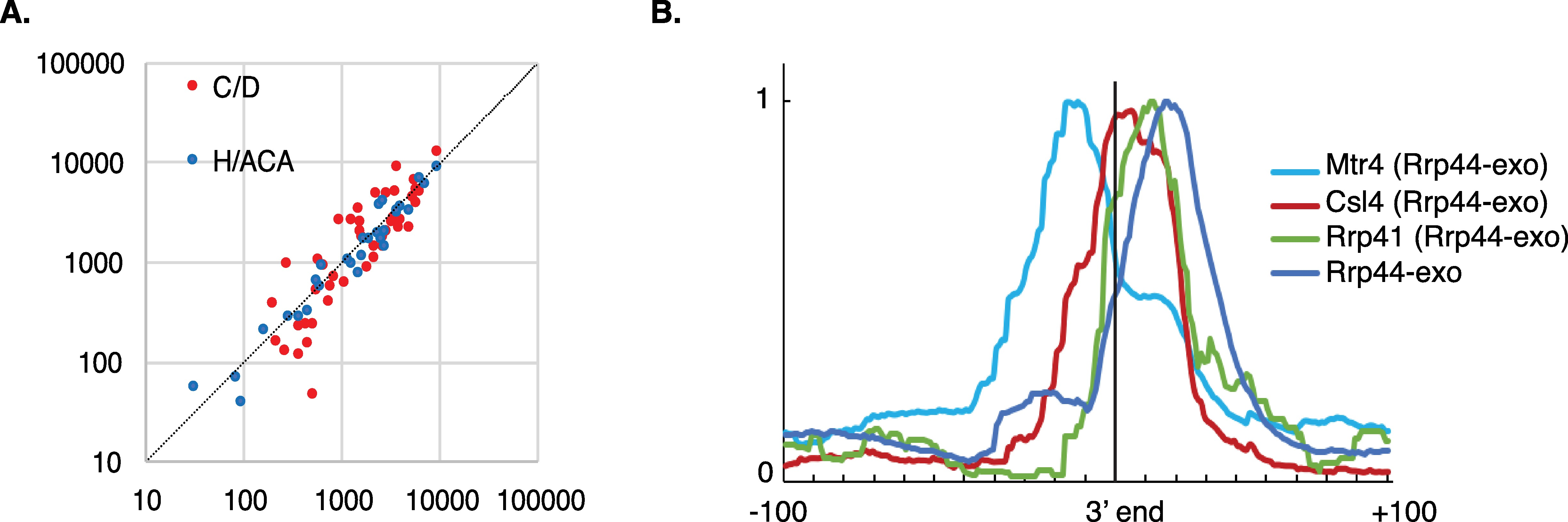

Supplement: S4 Fig — (A) RPKMs for each snoRNA species were averaged between two replicates of either Rrp44-exo or Rrp44-exo-S1 datasets and displayed on a 2D scatter plot. Box C/D and box H/ACA snoRNAs are represented in red and blue respectively. (B) Metagene analyses of all snoRNAs aligned by the 3’ end of the mature snoRNA region. Mtr4 (light blue), Csl4 (red), Rrp41 (green) in Rrp44-exo background, Rrp44-exo (blue) are shown. An average of two experiments was used for each sample, except for Rrp41 in which fewer reads were recovered and only the largest dataset is shown. (TIF) [file pgen.1006699.s004.tif]

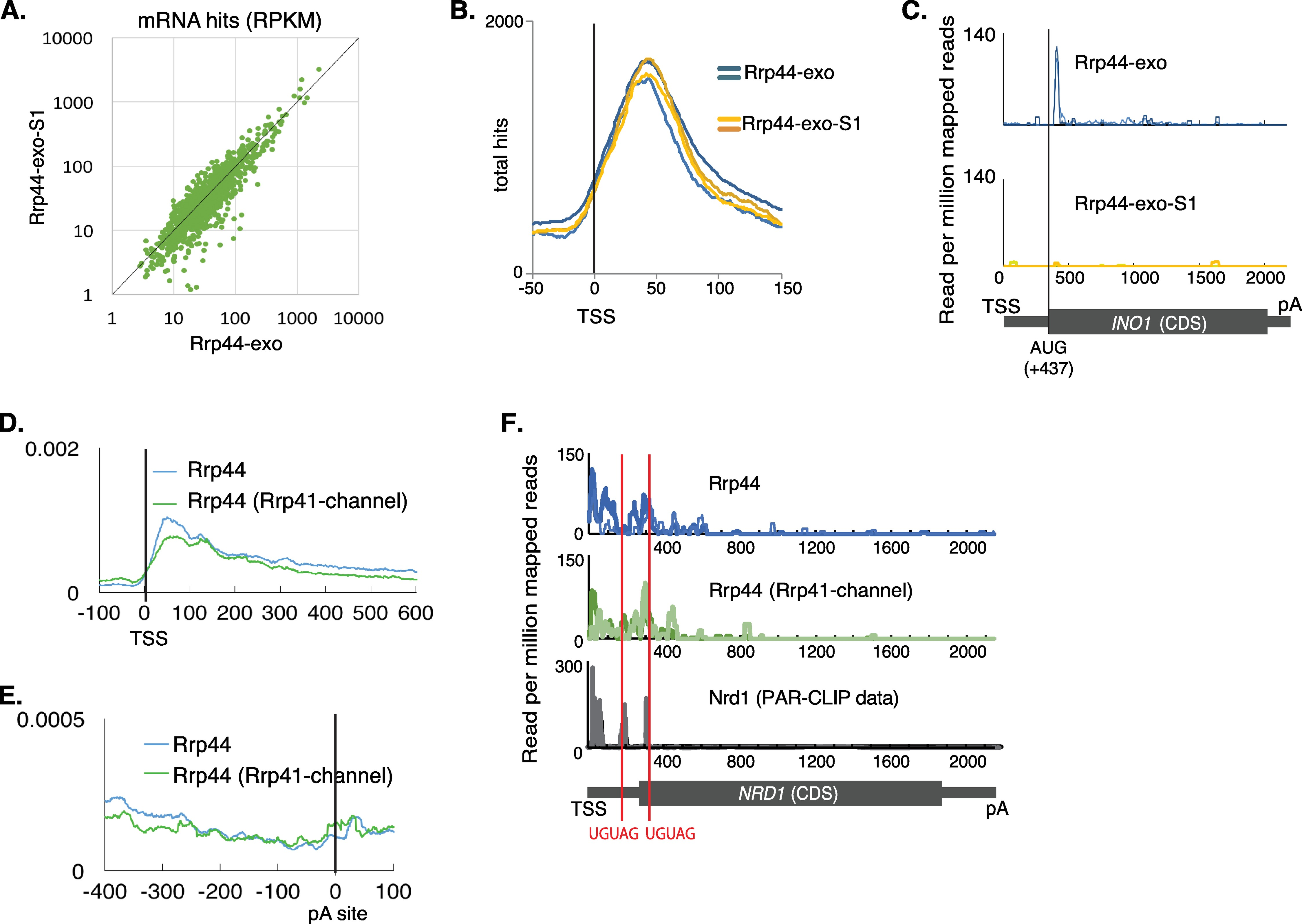

Supplement: S5 Fig — (A) RPKMs for each mRNA species were averaged between two replicates of either Rrp44-exo or Rrp44-exo-S1 datasets and displayed on a 2D scatter plot. (B) Metagene analyses of binding to top 1000 mRNAs aligned by TSS for Rrp44-exo (blue) and Rrp44-exo-S1 (yellow). Two independent experiments are shown for each analysis, normalized per million mapped reads. (C) Distribution of reads recovered with Rrp44-exo and Rrp44-exo-S1 across the INO1 gene, normalized by millions of mapped reads. Scale is linear. (D-E) Metagene analyses of binding of top 200 mRNA aligned by the TSS (D) or poly(A) site (E) for Rrp44 (blue) and Rrp44 (Rrp41-channel) (green). Data from two biological repeats were averaged for each strain background and represented as a fraction of total binding of Rrp44 across mRNAs for each strain. (F) Distribution of reads recovered with Rrp44, Rrp44 (Rrp41-channel) and Nrd1 [52] on NRD1, normalized by millions of mapped reads. Scale is linear. Locations of the consensus Nrd1-binding motifs (UGAUG) are also indicated. (TIF) [file pgen.1006699.s005.tif]
